# Role of 1,25-Dihydroxyvitamin D_3_ on Osteogenic Differentiation and Mineralization of Chicken Mesenchymal Stem Cells

**DOI:** 10.3389/fphys.2021.479596

**Published:** 2021-02-01

**Authors:** Chongxiao Chen, Roshan Adhikari, Dima Lynn White, Woo Kyun Kim

**Affiliations:** Department of Poultry Science, University of Georgia, Athens, GA, United States

**Keywords:** 1,25-dihydroxyvitamin D3, osteogenic differentiation, mineralization, osteogenic gene expression, chicken mesenchymal stem cells

## Abstract

1,25-dihydroxyvitamin D3 (1,25OHD) has been suggested to play an important role in osteogenic differentiation and mineralization. However, limited data have been reported in avian species. In the present study, the direct role of 1,25OHD on osteogenic differentiation and mineralization in chicken mesenchymal stem cells (cMSCs) derived from day-old broiler bones was investigated. cMSCs were treated with control media (C), osteogenesis media (OM), OM with 1, 5, 10, and 50 nM 1,25OHD, respectively. The messenger RNA (mRNA) samples were obtained at 24 and 48 h and 3 and 7 days to examine mRNA expression of key osteogenic genes [runt related transcription factor 2 (RUNX2), bone morphogenetic protein 2 (BMP2), collagen type I alpha 2 chain (COL1A2), bone gamma-carboxyglutamate protein (BGLAP), secreted phosphoprotein 1 (SPP1), and alkaline phosphatase (ALP)]. Cells were stained at 7, 14, and 21 days using Von Kossa (mineralization), Alizarin Red (AR; mineralization), and Alkaline Phosphatase (early marker) staining methods. From the mRNA expression results, we found a time-dependent manner of 1,25OHD on osteoblast differentiation and mineralization. In general, it showed an inhibitory effect on differentiation and mineralization during the early stage (24 and 48 h), and a stimulatory effect during the late cell stage (3 and 7 days). The staining showed 1,25OHD had an inhibitory effect on ALP enzyme activities and mineralization in a dosage-dependent manner up to 14 days. However, at 21 days, there was no difference between the treatments. This study provides a novel understanding of the effects of 1,25OHD on osteogenic differentiation and mineralization of cMSCs depending on cell stage and maturity.

## Introduction

Vitamin D_3_ requires two steps of hydroxylation to become 1,25-dihydroxyvitamin D_3_ (1,25OHD) to exert its biological functions (St-Arnaud, 2008). The effect of 1,25OHD is intricate and pleiotropic, which is involved in immunoregulation, anti-oxidation, anti-cancer actions, cardiovascular benefits, and bone development (Haussler et al., 2013; Gil et al., 2018). The role of vitamin D_3_ in bone development has been exhaustively studied in mammals (van Driel et al., 2004). It is well-established that 1,25OHD affects bone development either through the direct actions on bone cells or *via* regulating the mineral metabolism in the intestine, kidney, and parathyroid gland (Haussler et al., 2013; van Driel and van Leeuwen, 2014; Anderson, 2017; Gil et al., 2018). However, there are considerable disagreements of the direct effect of 1,25OHD in osteogenic differentiation and mineralization (St-Arnaud, 2008; Tarroni et al., 2012; van Driel and van Leeuwen, 2014). In human osteoblasts, 1,25OHD mostly showed stimulatory effects on osteogenic differentiation and mineralization (Prince et al., 2001; Chen et al., 2002; Jorgensen et al., 2004; Zhou et al., 2006; Tourkova et al., 2017; Li et al., 2018), but with a few exceptions (Viereck et al., 2002); In rat osteoblasts, the responses to 1,25OHD showed either inhibitory effects or no effects (Harrison et al., 1989; Kim and Chen, 1989); In mouse osteoblasts, the differentiation and mineralization of osteoblasts showed the most inconstant responses to 1,25OHD with either no effects, inhibition, or facilitation (van Driel and van Leeuwen, 2014; Chen et al., 2016; Kim et al., 2016; Xiong et al., 2017); Limited studies have been done in chicken osteoblasts, but the available data suggested, in general, that 1,25OHD showed an inhibitory effect on osteoblast differentiation and mineralization (Broess et al., 1995; Pande et al., 2015). These inconsistent results may be due to various experimental factors, such as species, cell stage, cell origin, treatment time, and dosage, and the presence of extracellular factors in each study (Czekanska et al., 2012; van Driel and van Leeuwen, 2014). Even though the role of vitamin D_3_ on osteoblast differentiation and mineralization has been comprehensively studied, questions regarding whether vitamin D_3_ has an anabolic or catabolic effect on osteoblasts and the role of vitamin D_3_ during different stages of cell growth and differentiation remain unanswered.

The chicken bone is an attractive bone research model due to its higher rate of mineralization (Pande et al., 2015) and unique bone turnover process (Whitehead, 2004). Owen et al. (1991) suggested that the effects of 1,25OHD on the expression of osteogenic differentiation markers could be affected by the basal expression level. In this case, chicken osteoblasts expressed higher osteocalcin and showed higher mineralization and alkaline phosphatase (ALP) expression compared to Rat-MSCs (Pande et al., 2015), providing an important insight contributing to understand the vitamin D_3_ function in bone formation.

In this study, chicken mesenchymal stem cells (cMSCs) isolated from compact bones of day-old broiler chicks were used to address the effects of various doses of 1,25OHD on osteogenic differentiation and mineralization of cMSCs during different cell stages. This study filled a research gap regarding roles of 1,25OHD on chicken osteogenic differentiation and mineralization.

## Materials and Methods

### Ethics Statement

All experiments were performed in accordance with the guidelines for the use of animal in research as stated by the Institutional Animal Care and Use Committee at the University of Georgia. The protocol was approved by the Institutional Animal Care and Use Committee at the University of Georgia.

### Isolation of cMSCs, Cell Culture, and Treatments

The isolation methods were adopted from Adhikari et al. (2019). In brief, legs of 1-day-old Cobb 500 male broiler chicks (three birds) were removed from the hip joint and soaked in alcohol for 2 min. The dissected legs were then kept in basal culture media [Dulbecco’s Modified Eagle’s medium (DMEM; Mediatech Inc., VA, United States) containing 10% Fetal Bovine Serum (FBS; Mediatech Inc., VA, United States), 100 U/ml penicillin, 100 μg/ml streptomycin, and 0.292 mg/ml L-glutamine (Thermo Fisher Scientific, MA, United States)]. Muscles and connective tissues around tibia and femurs were removed. The cleaned bones were placed in the washing buffer containing phosphate-buffered saline (PBS; Mediatech Inc., VA, United States) and 2% FBS. The epiphysis of the bone was removed, and bone marrow cavity was flushed with the washing buffer to remove the bone marrow and blood completely. The bones were then chopped to small fragments and suspended in digestion media [DMEM containing 100 IU/ml penicillin and 100 μg/ml streptomycin, 0.25% collagenase (Sigma-Aldrich, MO, United States), and 20% FBS]. Afterwards, the bone fragments were digested in an incubator shaker (Thermo Scientific SHKE4000, IA, United States) for 60 min at 37°C with the speed at 180 rpm. Then, the digestion media containing bone fragments were mixed with DMEM media containing 10% FBS in 1:2 ratio and filtered with 40 μm sterile filters. Filtered contents were centrifuged at 1,200 rpm for 10 min to concentrate the cells. The cells were pooled and resuspended with basal media and plated in 100-mm cell culture dishes (Falcon, NY, United States). Cultures were incubated at 37°C in a humidified incubator containing 5% CO_2_. The media were replaced every 2 days.

Upon the cells reached 95% confluency, the cells were washed twice with 5 ml 1X PBS (Mediatech Inc., VA, United States), then dissociated with 0.1% Trypsin-EDTA (Mediatech Inc., VA, United States) for 2 min and plated at a ratio of 25,000 cells/cm^2^ in a 100-mm cell culture dishes (Falcon, NY, United States). This passage was marked as P1; subsequent cultures were defined as P2, P3, P4… Pn consecutively. P4 cells were seeded at a density of 20,000 cells/cm^2^ in 24-well plates or 6-well plates (Falcon, NY, United States) for further testing. On confluency, the cells were treated with osteogenic media (OM) containing DMEM with 10^−7^ M dexamethasone (Sigma-Aldrich, MO, United States), 10 mM β-glycerophosphate (Sigma-Aldrich, MO, United States), 50 μg/ml ascorbate (Sigma-Aldrich, MO, United States), and 5% FBS for osteogenic induction. The treatments were OM with 1, 5, 10, and 50 nM 1,25OHD (Sigma-Aldrich, MO, United States), respectively. The cells cultured in DMEM basal media with 5% FBS were considered as a negative control. 1,25OHD were diluted in absolute grade ethanol (Sigma-Aldrich, MO, United States) to make the 1000x stock solution stored at −20°C upon treatment. All the other treatments were added with the same amount of ethanol to remove the vehicle regent’s potential effects. Fresh media were replaced every 2–3 days.

### Alkaline Phosphate Assay

Alkaline phosphatase activity was tested following a protocol for evaluating osteogenic differentiation of MSCs (PromoCell, Heidelberg, Germany). In brief, the cells were rinsed with PBS twice and fixed in 10% neutral buffered formalin (Sigma-Aldrich, MO, United States) for 60 s. Then the cells were washed with washing buffer containing 0.05% Tween 20 (Sigma-Aldrich, MO, United States) in PBS twice (Mediatech Inc., VA, United States), and 1 ml substrate solution [10 ml distilled water contained one SigmaFast™ BCIP/NBT tablet (Sigma-Aldrich, MO, United States)] was added to the wells. The cells were incubated at room temperature for 5–10 min based on color development. The reaction was stopped by rinsing the wells with PBS.

### Alizarin Red Staining

Alizarin red (AR) staining test was conducted to stain for the mineral deposition following the protocol defined by Gregory et al. (2004) to examine the mineralization level of cMSCs. In brief, Alizarin Red stain solution was prepared at the concentration of 20 mg/ml Alizarin Red S (Sigma-Aldrich, MO, United States) in distilled water at pH 4.1–4.3 (pH was adjusted by using 0.1% NH_4_OH). The cells were rinsed twice with PBS (without Ca^++^/Mg^++^), then fixed using 10% buffered formalin for 30 min, and washed twice with distilled water. The cells were stained with Alizarin Red solution for 45 min in the dark. The reaction was stopped by washing off the staining solution with distilled water.

### Von Kossa Staining

Von Kossa staining was performed for detecting mineralization following the protocol previously described by Parhami et al. (1997). In brief, the cells were rinsed with PBS and fixed with 0.1% glutaraldehyde (Sigma-Aldrich, MO, United States) for 15 min. Then, the cells were rinsed with distilled water and incubated with 5% silver nitrate (Sigma-Aldrich, MO, United States) in the dark for 30 min. The stained cells were rinsed again with distilled water and air dried under bright light to develop the color.

### RNA Extraction and Real-Time Quantitative Reverse Transcription PCR

Total RNA of cells was extracted by using QIAzol Lysis reagents (Qiagen, MD, United States) according to the manufacturer’s protocol. mRNA quantity and purity were determined (Nanodrop 1000 spectrophotometer, Thermo Fisher Scientific, Pittsburgh, PA). For each sample, 2 μg of RNA was reverse-transcribed to cDNA using a High-Capacity cDNA Reverse Transcription Kit (Thermo Fisher Scientific, MA, United States) following the manufacturer’s protocol in a 96-well thermal cycler (Thermo Fisher Scientific, MA United States). cDNA samples were analyzed in duplicate by quantitative Reverse Transcription PCR (qRT-PCR) using iTaq™ Universal SYBR Green Supermix (Bio-Rad, CA, United States). Primers (Table 1) and cDNA templates were subjected to qRT-PCR at 95°C for 10 min, followed by 40 cycles of 15 s denaturation at 95°C, annealing for 20 s (annealing temperature was varied depending on the primers; Table 1), and 15 s extension at 72°C, followed by 95°C for 15 s and a melt curve stage in a StepOneTM Real-Time PCR machine (Thermo Fisher Scientific, MA, United States). GAPDH was used as a housekeeping gene. Samples were normalized and analyzed by the *Δ*ΔCT method. All the primers in Table 1 were designed according to the NCBI gene sequence using Primer-BLAST.^1^

**Table 1:** List of primers used in this study.

Gene name	Primer sequence (5'---3')	Product length (bp)	Annealing temperature (°C)
GAPDH	Fwd: GCTAAGGCTGTGGGGAAAGT	116	56
	Rev: TCAGCAGCAGCCTTCACTAC		
RunX2	Fwd: TCTCTGAACTCTGCACCAAGTC	229	58
	Rev: GCTCGGAAGCACCTGAGAGG		
Col1A2	Fwd: CTGGTGAAAGCGGTGCTGTT	222	57
	Rev: CACCAGTGTCACCTCTCAGAC		
SPP1	Fwd: GCCCAACATCAGAGCGTAGA	204	57
	Rev: ACGGGTGACCTCGTTGTTTT		
BMP2	Fwd: TCAGCTCAGGCCGTTGTTAG	163	57
	Rev: GTCATTCCACCCCACGTCAT		
BGLAP	Fwd: GGATGCTCGCAGTGCTAAAG	142	57
	Rev: CTCACACACCTCTCGTTGGG		
ALP	Fwd: CGACCACTCACACGTCTTCA	140	58
	Rev: CGATCTTATAGCCAGGGCCG		

### Statistics

All experimental data were analyzed statistically by one-way ANOVA using SAS software Version 9.3 (SAS Institute, Cary, NC). Variability in the data was expressed as SEM. Differences between means were determined using Duncan’s Multiple Range test. The level of significance was assessed at *p* ≤ 0.05.

## Results

### Key Osteogenic Differentiation Marker Gene Expression

**Figure 1:**
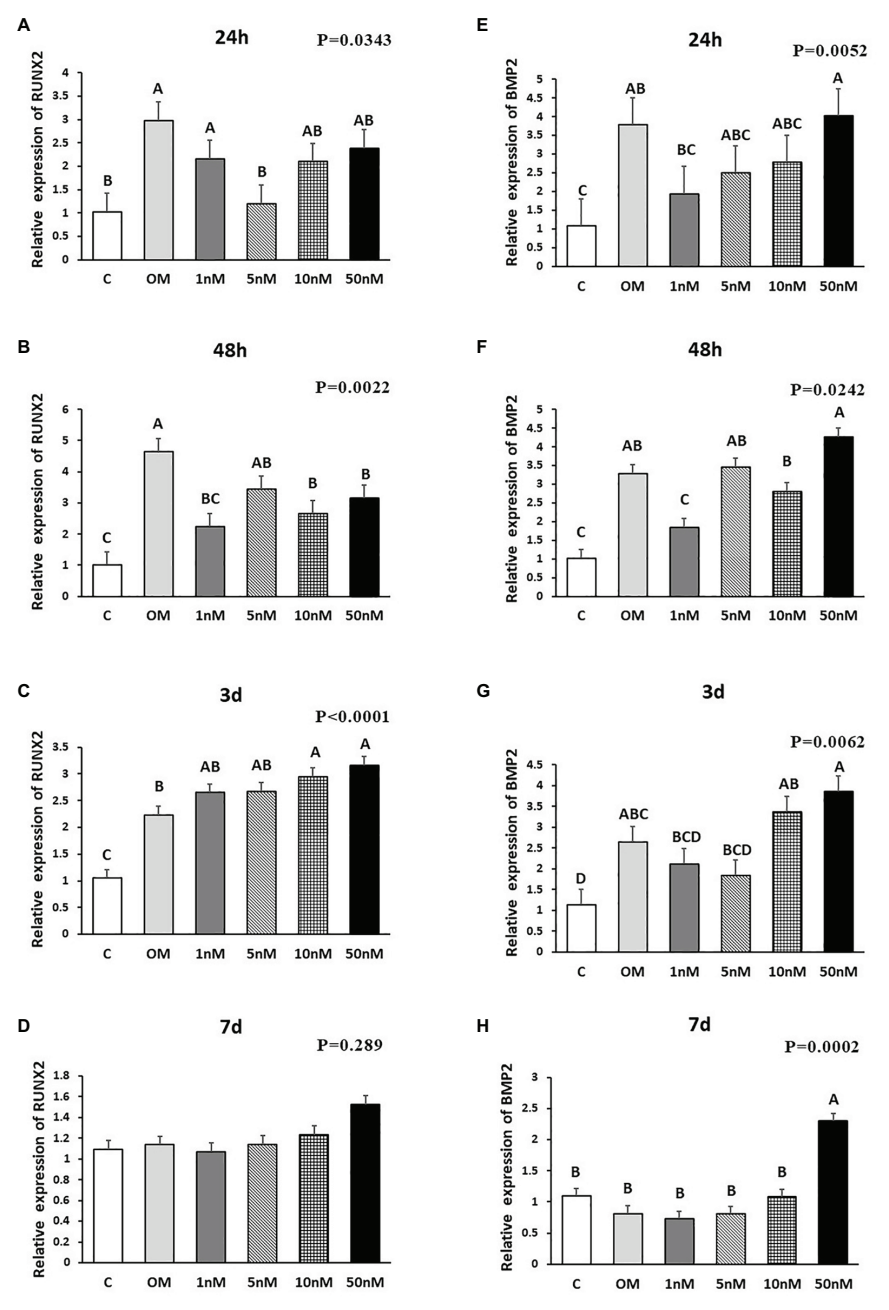
Effects of 1,25-Dihydroxyvitamin D_3_ (1,25OHD) on chicken osteoblasts runt related transcription factor 2 (RUNX2) and bone morphogenetic protein 2 (BMP2) gene expression by using quantitative Reverse Transcription PCR (qRT-PCR). Cells were treated with control media, osteogenesis media, or osteogenesis media with 1, 5, 10, or 50 nM 1,25OHD. **(A–D)** showed the messenger RNA (mRNA) expression of RUNX2 at 24 and 48 h and 3 and 7 days, respectively; **(E–H)** showed the mRNA expression of BMP2 at 24 and 48 h and 3 and 7 days, respectively. **(A–D)** Mean separation was indicated by different letters on the top of bars (Value means ± SEM, three replicates/treatment).

**Figure 2:**
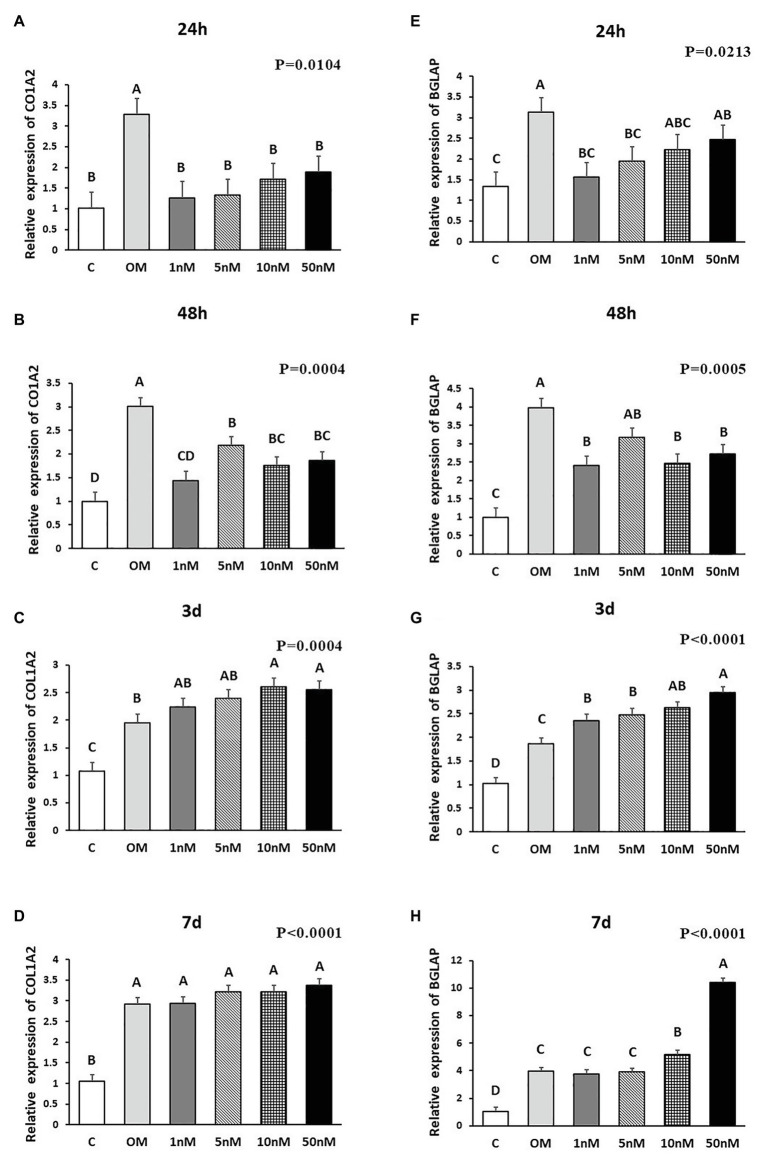
Effects of 1,25OHD on chicken osteoblasts collagen type I alpha 2 chain (COL1A2) and bone gamma-carboxyglutamate protein (BGLAP) gene expression by using qRT-PCR. Cells were treated with control media, osteogenesis media, or osteogenesis media with 1, 5, 10, or 50 nM 1,25OHD. **(A–D)** showed the mRNA expression of COL1A2 at 24 and 48 h and 3 and 7 days, respectively; **(E–H)** showed the mRNA expression of BGLAP at 24 and 48 h and 3 and 7 days, respectively. **(A–D)** Mean separation was indicated by different letters on the top of bars (Value means ± SEM, three replicates/treatment).

**Figure 3:**
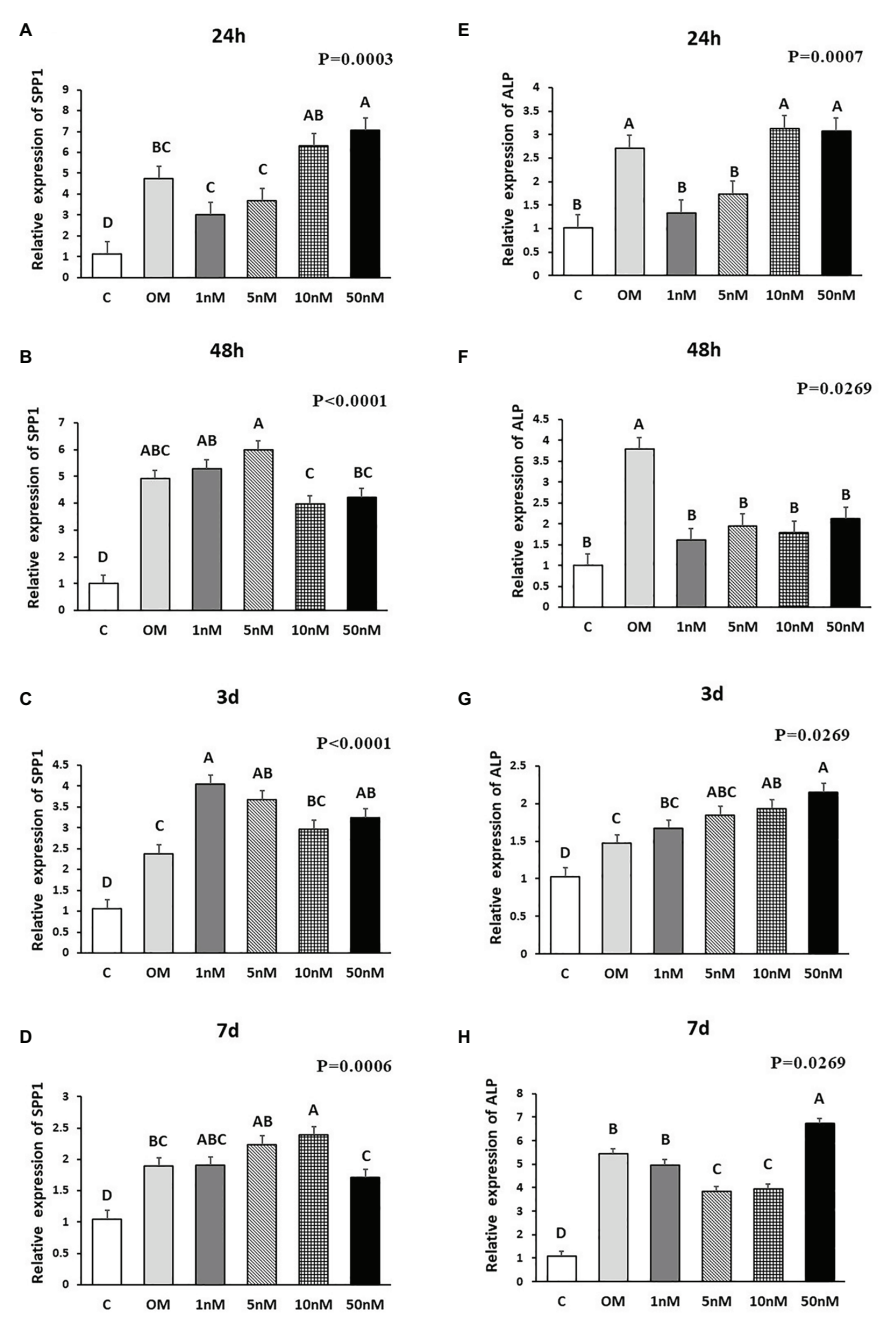
Effects of 1,25OHD on chicken osteoblasts secreted phosphoprotein 1 (SPP1) and alkaline phosphatase (ALP) gene expression by using qRT-PCR. Cells were treated with control media, osteogenesis media, or osteogenesis media with 1, 5, 10, or 50 nM 1,25OHD. **(A–D)** showed the mRNA expression of SPP1 at 24 h, 48 h, 3 days, and 7 days, respectively; **(E–H)** showed the mRNA expression of ALP at 24 h, 48 h, 3 days, and 7 days, respectively. **(A–D)** Mean separation was indicated by different letters on the top of bars (Value means ± SEM, three replicates/treatment).

In order to study the role of 1,25OHD in chicken osteogenic differentiation of cMSCs, the samples were obtained at 24 and 48 h and 3 and 7 days post-treatment for the assessment of key osteogenic gene expression (RUNX2: runt related transcription factor 2; BMP2: bone morphogenetic protein 2, COL1A2: collagen type I alpha 2 chain; BGLAP: bone gamma-carboxyglutamate protein; SPP1: secreted phosphoprotein 1; and ALP: alkaline phosphatase). The gene expression results are shown in Figures 1–3. OM induced mRNA expression of key osteogenic genes at all time points compared to control (C; *p* < 0.05), except for RUNX2 and BMP2 at 7 days (Figures 1–3), which confirmed that the experimental conditions were adequate to induce osteogenic differentiation in cMSCs. RUNX2 expression was inhibited by 1, 10, and 50 nM 1,25OHD treatments at 48 h compared to OM treatment (*p* = 0.002; Figure 1B). In contrast, 1,25OHD upregulated RUNX2 expression at 3 days with the 10 and 50 nM 1,25OHD compared with OM treatment (*p* < 0.0001), and there was a tendency for 1,25OHD to increase its expression as a dose-response manner (Figure 1C), although no significant difference in RUNX2 expression was found at 7 days among the treatments (Figure 1D). For BMP2 expression, at 48 h, only the 1 nM 1,25OHD inhibited its expression compared to OM treatment (*p* = 0.024; Figure 1F). However, no significant difference was found at 24 h and 3 days compared with OM treatment (Figures 1E,G). The highest dose of 1,25OHD had significantly higher BMP2 expression compared to 1, 5, or 10 nM 1,25OHD at 24 h, 48 h, or 3 days (Figures 1E–G). Furthermore, the 50 nM 1,25OHD significantly stimulated BMP2 expression at 7 days (*p* < 0.001) compared to the other treatments (Figure 1H).

**Figure 4:**
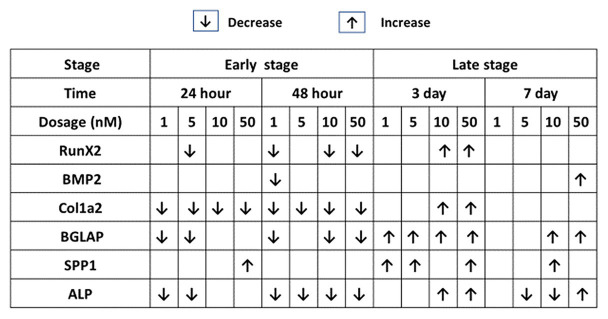
Effects of 1,25OHD on chicken osteoblasts key osteogenic gene expression by using qRT-PCR. Cells were treated with control media, osteogenesis media, or osteogenesis media with 1, 5, 10, or 50 nM 1,25OHD. The results are summarized in the figure: the down-arrows indicate the genes are down-regulated by 1,25OHD treatment compare to OM; the up-arrows indicate the genes are upregulated by 1,25OHD treatment compare to OM (Value means ± SEM, three replicates/treatment).

The effects of 1,25OHD on bone matrix protein mRNA expression followed a similar pattern as RUNX2 and BMP2. All concentrations of 1,25OHD decreased COL1A2 expression at the earlier time points (24 and 48 h) compared to OM treatment (*p* < 0.05; Figures 2A,B). On the contrary, at 3 days, 10 and 50 nM 1,25OHD increased COL1A2 expression compared to OM treatment (*p* < 0.0004; Figure 2C). At 7 days, no significant differences in COL1A2 expression were observed between 1,25OHD and OM treatments, whereas OM and 1,25OHD treatments significantly enhanced its expression compared to control (*p* < 0.0001). BGLAP showed similar expression response to 1,25OHD as COL1A2 (*p* < 0.05). At early time points, the 1,25OHD treatment decreased BGALP expression except 10 and 50 nM dosage at 24 h and 5 nM dosage at 48 h (Figures 2E,F). However, at late time points, 1,25OHD increased BGLAP expression except 1 and 5 nM dosage levels at 7 days (Figures 2G,H). SPP1 was increased by 50 nM 1,25OHD at 24 h (*p* = 0.0003) compared to OM treatment (Figure 3A), whereas 50 nM 1,25OHD decreased SPP1 expression at the later time points (48 h, 3 days, and 7 days) compared to the lower dose treatments (1, 5, or 10 nM; Figures 3B–D); the lower dose treatments (1 and 5 nM) started lower SPP1 expression at 24 h but then increased its expression at the later time points (48 h, 3 days, or 7 days). The increased SPP1 by 10 nM 1,25OHD was found at 7 days compared to OM treatment (*p* = 0.0006; Figure 3D). The ALP expression response to 1,25OHD was found in the similar pattern as COL1A2 at 24 h, 48 h, and 3 days except 10 and 50 nM 1,25OHD at 24 h (*p* < 0.05); 1 and 5 nM 1,25OHD inhibited ALP expression induced by OM treatment, whereas 10 and 50 nM 1,25OHD had no inhibitory effects on ALP expression at 24 h (Figures 3E–G). All 1,25OHD treatments inhibited ALP expression compared to OM treatment at 48 h, but they increased its expression at 3 days. During the late stage of differentiation, 1,25OHD showed a dual function on ALP expression; at 7 days, 5 and 10 nM 1,25OHD suppressed ALP expression, whereas 50 nM increased ALP expression (*p* < 0.05; Figure 3H). In order to better visualize the overall effects of 1,25OHD on osteogenic differentiation of cMSCs, the gene expression results are summarized in Figure 4. Overall effects of 1,25OHD on osteogenic differentiation of cMSCs were inhibitory at the earlier stages (24 and 48 h) and stimulatory at the later stages (3 and 7 days).

### ALP Activity and Mineralization

**Figure 5:**
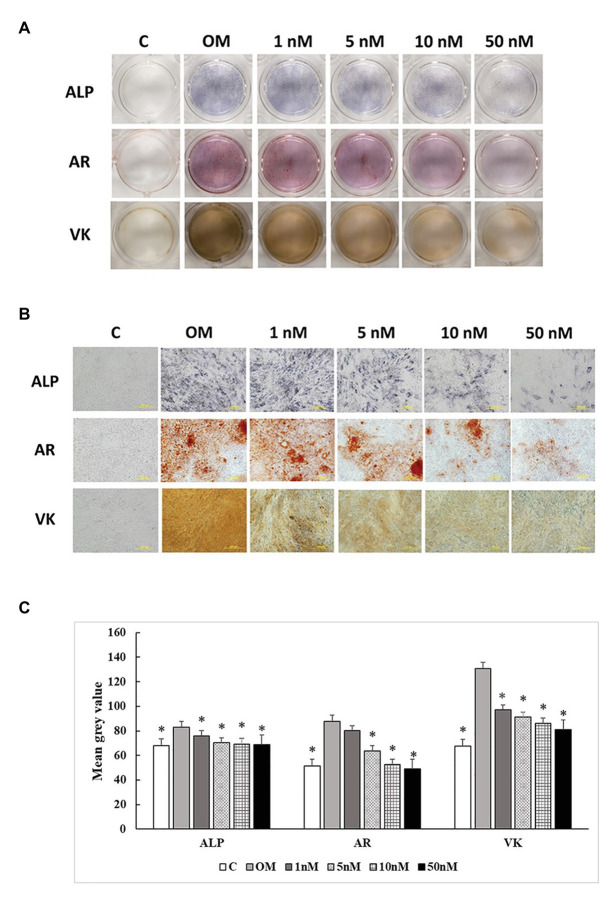
Effects of 1,25OHD on chicken osteoblasts ALP activity and mineralization at 7 days. In each picture, the first row: ALP (Alkaline Phosphatase); the second row: AR (Alizarin Red stains the deposited minerals); and the third row: VK (Von Kossa stains for the deposited minerals). **(A)** Pictures were taken by DSRL camera, with same exposure setting across all the treatment. **(B)** The picture was taken under the microscope at 40x. The bar indicated 200 μm. **(C)** The pictures (40x) were invert and converted to 8-bit gray scale using Image J software (1.48v version, U. S. National Institutes of Health, Bethesda, MD, United States). The mean gray value (0.16 mm^2^ area) was measured. The values were presented as mean gray value. The higher value represents darker staining (Value means ± SEM, three replicates/treatment; ^*^means significant difference compared to OM; ALP: *p* < 0.0001; AR: *p* < 0.0001; VK: *p* < 0.0001).

**Figure 6:**
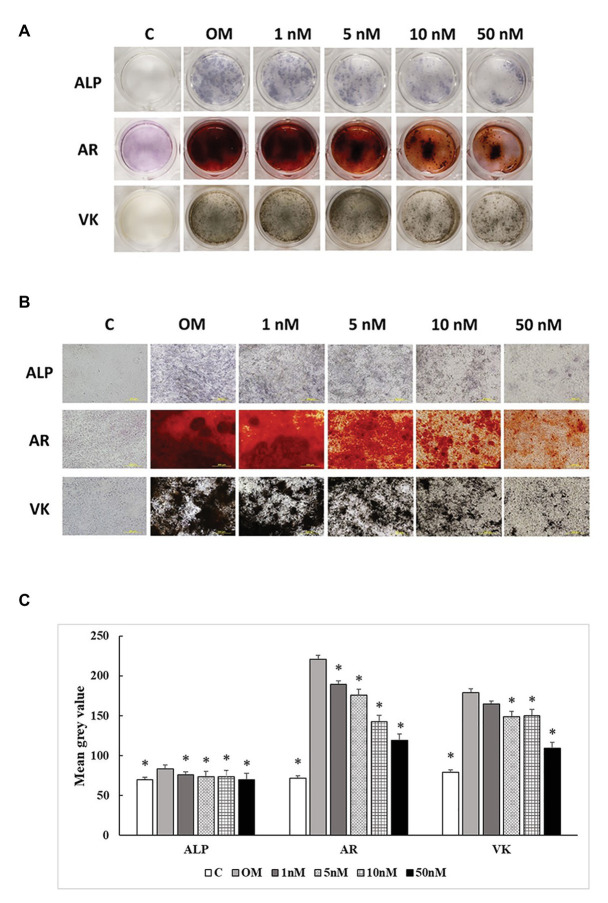
Effects of 1,25OHD on chicken osteoblasts ALP activity and mineralization at 14 days. In each picture, the first row: ALP (Alkaline Phosphatase); the second row: AR (Alizarin Red stains the deposited minerals); and the third row: VK (Von Kossa stains for the deposited minerals). **(A)** Pictures were taken by DSRL camera, with same exposure setting across all the treatment. **(B)** The picture was taken under the microscope at 40x. The bar indicated 200 μm. **(C)** The pictures (40x) were invert and converted to 8-bit gray scale using Image J software (1.48v version, U. S. National Institutes of Health, Bethesda, MD, United States). The mean gray value (0.16 mm^2^ area) was measured. The values were presented as mean gray value. The higher value represents darker staining (Value means ± SEM, three replicates/treatment; ^*^means significant difference compared to OM; ALP: *p* = 0.0022; AR: *p* < 0.0001; VK: *p* < 0.0001).

**Figure 7:**
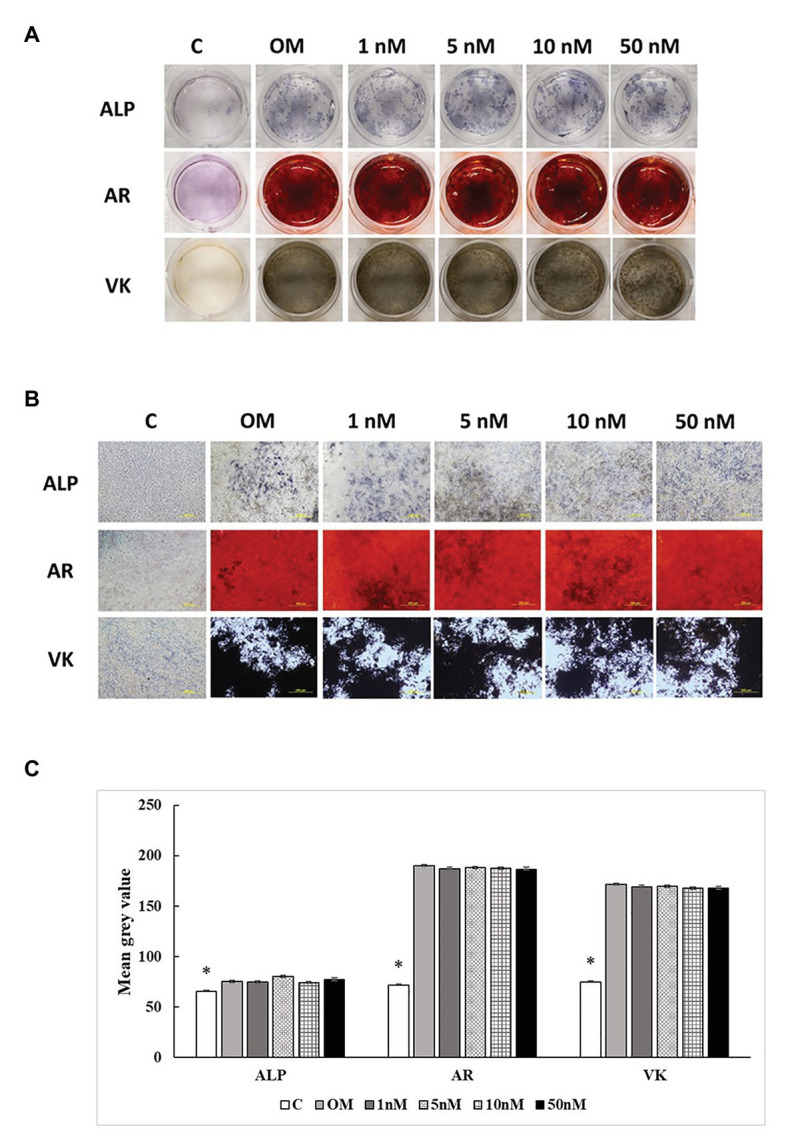
Effects of 1,25OHD on chicken osteoblasts ALP activity and mineralization at 21 days. In each picture, the first row: ALP (Alkaline Phosphatase); the second row: AR (Alizarin Red stains the deposited minerals); and the third row: VK (Von Kossa stains for the deposited minerals). **(A)** Pictures were taken by DSRL camera, with same exposure setting across all the treatment. **(B)** The picture was taken under the microscope at 40x. The bar indicated 200 μm. **(C)** The pictures (40x) were invert and converted to 8-bit gray scale using Image J software (1.48v version, U. S. National Institutes of Health, Bethesda, MD, United States). The mean gray value (0.16 mm^2^ area) was measured. The values were presented as mean gray value. The higher value represents darker staining (Value means ± SEM, three replicates/treatment; ^*^means significant difference compared to OM; ALP: *p* = 0.0447; AR: *p* < 0.0001; VK: *p* < 0.0001).

Three staining methods were performed at 7, 14, and 21 days to explore the ALP activity and mineralization process of cMSCs. The staining results were presented in two formats. The whole well staining pictures were taken using a DSLR camera with a fixed exposure setting for all the treatments (Figures 5A, 6A, 7A). At the same time, the staining pictures were taken by a microscope at 40x magnificent (Figures 5B, 6B, 7B). As the osteogenic differentiation of cMSCs was progressed, the mineralization level became higher on the cells. While comparing the treatments at each time point, OM treatment dramatically increases the ALP activity (ALP staining) and mineralization (Von Kossa and Alizarin Red staining) compared with control at all time points (Figures 5C, 6C, 7C). For 1,25OHD treatments, at 7 days, the cells with 1,25OHD treatments showed inhibitory effects on ALP activity and mineralization, which are evidenced by less staining was observed ALP (*p* < 0.0001), Alizarin Red (AR, *p* < 0.0001), and Von Kossa staining (VK, *p* < 0.0001) compared to OM treatment (Figure 5C). But there is no difference observed in AR staining while comparing 1 nM 1,25OHD treatment to OM. Similarly, at 14 days, the cells treated with 1,25OHD showed reduced ALP activity (*p* = 0.0022) and decreased mineralization (AR: *p* < 0.0001; VK: *p* < 0.0001) compared to the cells treated with OM, whereas no difference was observed in 1 nM 1,25OHD treatment compared to OM regarding VK staining. However, at 21 days, no differences between OM and 1,25OHD treatments were found in all three types of staining (Figure 6C). Furthermore, higher concentrations of 1,25OHD showed a trend of stronger inhibitory effects in ALP activity and mineralization at both 7 and 14 days (Figures 4, 5).

## Discussion

1,25-dihydroxyvitamin D3 is regarded as a regulator of bone metabolism by affecting the production of bone matrix proteins, the activity of alkaline phosphatase, and the process of mineralization in bone (Yamamoto et al., 2013). However, its function on osteoblasts is intricated due to many factors involved, such as species, cell type, origin, and stage, treatment timing, and the presence of extracellular factors (St-Arnaud, 2008; Yang et al., 2013; van Driel and van Leeuwen, 2014). It is challenging to include all the factors in one study. Hence, this study mainly focused on the effects of various doses of 1,25OHD on osteogenic differentiation of cMSCs during different differentiation stages.

In the current study, we demonstrated a time-dependent manner of 1,25OHD on the expression of key osteogenic differentiation marker genes. 1,25OHD showed inhibitory effects during the early differentiation stages (24 and 48 h), but stimulatory effects during the late differentiation stages (3 and 7 days). Limited research has paid attention to such time-related function. Broess et al. (1995) mentioned a similar timing related function in a chicken osteoblasts study, which indicated 10 nM of 1,25OHD slightly stimulated osteopontin and osteocalcin expression at the 17 days but showed the inhibitory effects at 5 and 30 days. In rat osteoblasts, the acute 1,25OHD treatment inhibited alkaline phosphatase and collagen I expression in the early phase but stimulated expression during the mineralization period (Owen et al., 1991). These results suggest that 1,25OHD may play different roles at various osteogenic differentiation stages. The additional proves can be found in a human osteoblast study reporting that pre-mineralization (before 10 days) and mineralization phases (after 10 days) of human osteoblasts are two distinctive periods, and there is only 0.6% (three genes) overlap of genes regulated by 1,25OHD (Woeckel et al., 2010). Mostly likely, 1,25OHD has different mechamisms on regulating differentiation and mineralization in osteoblasts.

Among these osteogenesis markers, RUNX2 as one of the most important osteoblast-specific transcription factors is essential for the activation of osteoblast differentiation and the induction of MSCs into mature osteoblasts (Mundlos et al., 1997; Viereck et al., 2002; Komori, 2006; Lieben et al., 2012; Vimalraj et al., 2015). Similarly, BMP2 is highly related to the induction of osteogenic differentiation and enhancement of bone matrix production (Zhou et al., 2006; Liu et al., 2012). However, in the current study, 1,25OHD had a tendency to inhibit RUNX2 and BMP2 expression with a dose-specific manner at the earlier stages of the differentiation (24 and 48 h), which is similar to the study in mouse osteoblasts (Ducy et al., 1997; Yang et al., 2013; Kim et al., 2016). For human osteoblasts, the responses are more complicated due to the 1,25OHD treatment duration and cell lines. The treatment duration of 1,25OHD affects its function on RUNX2 expression in human primary osteoblasts with a short 1 h treatment showed stimulatory effects, whereas 48 h treatment showed an inhibitory effect (Viereck et al., 2002). Furthermore, various human osteoblast cell lines exhibited different responses to 1,25OHD; RUNX2 was not affected by 1,25OHD in human osteoblasts cell lines (O4-T8, 03-CE6, and 03-CE10; Prince et al., 2001), whereas it was upregulated by 1,25OHD in human marrow stromal cells (Geng et al., 2011). Similarly to RUNX2, BMP2 is either increased or decreased by 1,25OHD across species (Chatakun et al., 2014). The effects of 1,25OHD in progenitor cells and osteoblasts are complicated. However, the fact that cells in each study display varied levels of the responses depending on differentiation stages, aligns with our hypothesis that different responses of 1,25OHD in osteoblasts may be related to cell differentiation stages. In the current study, we carefully investigated 1,25OHD actions at various time points and dosage, which reveals that cell differentiation stage and dosage are important for chicken osteogenic differentiation and mineralization.

Most of osteogenic markers, such as COL1A2 and BGLAP, followed the time-dependent manner as described above except SPP1 and ALP. SPP1 was stimulated at 24 h with the highest treatment dosage, which is the only exception for all the inhibitory effects observed at this stage. Whereas, besides being an osteoblast differentiation marker, SPP1 can also inhibit mineralization (Chabas, 2005). Therefore, the higher expression of SPP1 at 24 h in the current study may potentially act as an inhibitory factor for bone mineralization at early cell stage. The ALP gene expression was the only tested gene that was downregulated by 5 and 10 nM 1,25OHD at 7 days, which agrees with previous mouse studies (Chen et al., 2012; Kim et al., 2016). However, the ALP expression is upregulated by 50 nM 1,25OHD in the currently study. Similar changes were found in human osteoblasts, where 1,25OHD either showed no effects or promoting effects on ALP expression (Matsumoto et al., 1991; Siggelkow et al., 1999; Chen et al., 2002). There is one possible explanation for these variations; the multiple functions of bone matrix proteins may contribute to this complexity.

The staining results from this study showed the inhibitory effects on ALP activity and mineralization up to 14 days, but no difference was observed at 21 days, which indicates that the stimulatory effects after 3 days balanced off the earlier inhibitory effects by 1,25OHD. Contrary to our finding, in human osteoblasts, Prince et al. (2001) found the continuous treatment with 1,25OHD inhibited mineralization of the 04-T8 and CE6 cell lines but promoted mineralization in the CE10 cell line, indicating that CE10 reflects an earlier stage osteoblasts phenotype than CE6 and O4-T8. Yang et al. (2013) also pointed out the different mineralization response of calvaria and long bone osteoblasts to 1,25OHD are because of different maturation level of two cell origins. Regardless the varied results in our current and previous other studies, these studies all indicated the effect of 1,25OHD on osteogenic differentiation and mineralization is related to the cell mineralization stage and maturity. There were no differences found in staining at 21 days. It is probably because the mineralization has reached saturation due to a much higher mineralization level observed from chicken osteoblasts than other research models, such as Rat (Pande et al., 2015).

In the current study, we found that the effects of 1,25OHD on osteogenic differentiation of cMSCs are differentiation stage and dosage specific manners. However, in order to translate such understanding of 1,25OHD on cMSCs into whole chicken model, we need to explore the interaction of osteoblast and osteoclast under the treatment of 1,25OHD and bone metabolism contributed by other organs, such as intestine, kidney, and parathyroid (Haussler et al., 2013). This fascinating time-dependent manner may positively affect bone remodeling process in chicken. The bone remodeling is initiated by osteoclastic bone resorption, which erodes a resorption lacuna, then followed by bone formation (Eriksen, 2010). Even though, such a remodeling event may have significant overlaps during different remodeling stages (Schindeler et al., 2008). Nevertheless, the time interval between the activation of osteoblasts and osteoclasts may enhance the efficiency of the bone remodeling process. The 1,25OHD inhibitory effects during the early differentiation stage of osteoblasts may be a sign of creating time interval during bone remodeling. However, due to the complexity of this process, it still requires more studies to further explore this theory.

In summary, this study emphasizes that effects of 1,25OHD are greatly related to the cell differentiation stage in chicken osteoblasts. 1,25OHD showed inhibitory effects on cMSC osteogenic differentiation and mineralization during the early cell stage but stimulatory effects during the late cell stage. Furthermore, the staining results indicate a dosage-dependent manner; the higher levels of 1,25OHD showed stronger inhibitory effects on mineralization of cMSCs up to 14 days. However, the detailed mechanism is still necessary to be eluciated. A number of factors may contribute to the complexity of 1,25OHD effects on osteoblasts. Thus, the interpretation of data related to 1,25OHD on bone cells needs to address the specific factors, such as species, cell, type, cell origin, differentiation stage, extracellular factor, treatment dosage, duration, etc. Hence, systematic evaluation of the interaction among these factors is important while eluciating effects of 1,25OHD on osteoblast differentiation and mineralization.

## Data Availability Statement

All datasets generated for this study are included in the article/supplementary material.

## Ethics Statement

The animal study was reviewed and approved by the Institutional Animal Care and Use Committee at the University of Georgia.

## Author Contributions

All authors listed have made a substantial, direct and intellectual contribution to the work, and approved it for publication. WK conceived and designed this study. CC and RA contributed to isolation of cBMSCs, cell differentiation, qRT-PCR, staining, and data analyses. The paper was written through contribution and critical review of the manuscript by CC, RA, DW, and WK.

### Conflict of Interest

The authors declare that the research was conducted in the absence of any commercial or financial relationships that could be construed as a potential conflict of interest.

## Footnotes

^1^https://www.ncbi.nlm.nih.gov/tools/primer-blast/index.cgi
